# Combined enhancement of the propionyl-CoA metabolic pathway for efficient androstenedione production in *Mycolicibacterium neoaurum*

**DOI:** 10.1186/s12934-022-01942-x

**Published:** 2022-10-20

**Authors:** Zhenhua Su, Zhenjian Zhang, Jian Yu, Congcong Yuan, Yanbing Shen, Jianxin Wang, Liqiu Su, Min Wang

**Affiliations:** 1 Key Laboratory of Industrial Fermentation Microbiology, Ministry of Education, Tianjin Key Laboratory of Industrial Microbiology, College of Biotechnology, Tianjin University of Science & Technology, Tianjin, 300457 China; 2 Frontage Laboratories, Inc, Exton, PA 19341 USA

**Keywords:** *Mycolicibacterium neoaurum*, Androstenone, Propionyl-CoA metabolic, Pathway Associates

## Abstract

**Background:**

The production of androstenedione (AD) from phytosterols by *Mycolicibacterium neoaurum* is a multi-step biotransformation process, which requires degradation of sterol side chains, accompanied by the production of propionyl-CoA. However, the transient production of large amounts of propionyl-CoA can accumulate intracellularly to produce toxic effects and severely inhibit AD production.

**Results:**

In the present study, the intracellular propionyl-CoA concentration was effectively reduced and the productivity of the strain was improved by enhancing the cytosolic methyl-branched lipid synthesis pathway and increasing the expression level of *nat* operator gene, respectively. Subsequently, the application of a pathway combination strategy, combined and the inducible regulation strategy, further improved AD productivity with a maximum AD conversion rate of 96.88%, an increase of 13.93% over the original strain.

**Conclusions:**

Overall, we provide a new strategy for reducing propionyl-CoA stress during biotransformation for the production of AD and other steroidal drugs using phytosterols.

**Supplementary Information:**

The online version contains supplementary material available at 10.1186/s12934-022-01942-x.

## Introduction

Androstenedione (AD) is an important steroid intermediate, and almost all steroid drugs such as adrenocorticotropic hormones, sex hormones and anabolic hormones can be produced from AD [1], and more than 100 kinds of related steroid hormone drugs have been synthesized from AD in the market, which has a broad market [2]. Microbial conversion of phytosterols for AD production is an environmentally friendly and low-cost production method to successfully replace the traditional chemical synthesis method [3]. *Mycolicibacterium* spp. are the main AD-producing genera due to their powerful sterol degradation system [4].

In the process of AD production by *Mycolicibacterium neoaurum* (MNR) using phytosterols as substrate, the initial oxidation and side-chain degradation will produce a large amount of propionyl-CoA in a short period of time, which makes the strain unable to degrade it rapidly through normal metabolism, thus leading to excessive accumulation of propionyl-CoA in the cell and thus inhibiting the growth of the bacterium [5–7]. Three propionyl-CoA metabolic pathways exist in *Mycolicibacterium* spp.: the 2-methylcitrate cycle pathway (MCC), the methylmalonyl cycle pathway (MMC) [5], and the cytosolic methyl-branched lipid synthesis pathway [5, 8]. Previous studies succeeded in reducing the intracellular propionyl CoA content by enhancing the first two pathways, ultimately resulting in a 28.4% increase in transformation efficiency compared to the original strain [6, 7].

High concentrations of substrate (phytosterols) and product (AD) during AD production can be toxic to microorganisms [9–11]. In *Mycobacterium tuberculosis* (MTB), through the cytosolic methyl-branched lipid synthesis pathway, propionyl-CoA /acetyl-CoA carboxylase (ACC) catalyzes the intracellular malonyl-CoA and acetyl-CoA to methylmalonyl-CoA and malonyl-CoA [12, 13]. Later, in the presence of polyketide synthase (PikA1), the two undergo an esterification condensation reaction to produce nabolone, which is an important component in the synthesis of the cytosolic methyl-branched lipid phthiocerol dimycocerates (PDIM) [14, 15]. When MTB infect the host, in order to improve its tolerance in the host, they produce sufficient acetyl-coenzyme A and malonyl coenzyme A by absorbing cholesterol from the host, and later express the ACC family genes at high levels to produce malonyl coenzyme A and methyl malonyl coenzyme A. The two, in the presence of a polyketide synthase complex, generate PDIM, which improves the tolerance of the strain [16]. Therefore, the cytosolic methyl-branched lipid synthesis pathway has the potential to be a potentially effective way to effectively reduce intracellular propionyl-CoA content and enhance the tolerance of the bacterium, however, there are few studies on the use of the cytosolic methyl-branched lipid synthesis pathway for AD production in MNR to date.

In addition to this, another potential propionyl-CoA metabolic pathway emerged in our view. In MTB and *Mycolicibacterium smegmatis*, there is a *nat* operon that encodes the Hsa family protein HsaA-D and N-acetyltransferase (NAT) protein that are directly involved in the cholesterol degradation pathway of MTB [17], where NAT uses acyl-coenzyme A to degrade cholesterol catabolic metabolites, such as acetyl-CoA and propionyl-CoA, to enrich the cellular acyl pool for normal life activities such as cell growth [18, 19]. In *Mycolicibacterium smegmatis nat* operon, expression of the hsaA-D gene activates the transcription factor AraC. *Nat* gene is regulated by AraC, which recognizes free amino groups on various intracellular arylamine and hydrazine structural scaffolds, and catalyzes the transfer of acetyl groups from acetyl-CoA to the arylamine scaffold, releasing free CoA and re-producing acetyl-CoA, succinyl-CoA, propionyl-CoA and malonyl-coenzyme A, etc. [20]. It has been shown that increasing intracellular coenzyme A levels is effective in improving cell viability and thus the accumulation of target products [21, 22]. However, whether the action of this operon is beneficial for AD production by *Mycolicibacterium* spp., to our knowledge, has not been reported.

**Fig. 1:**
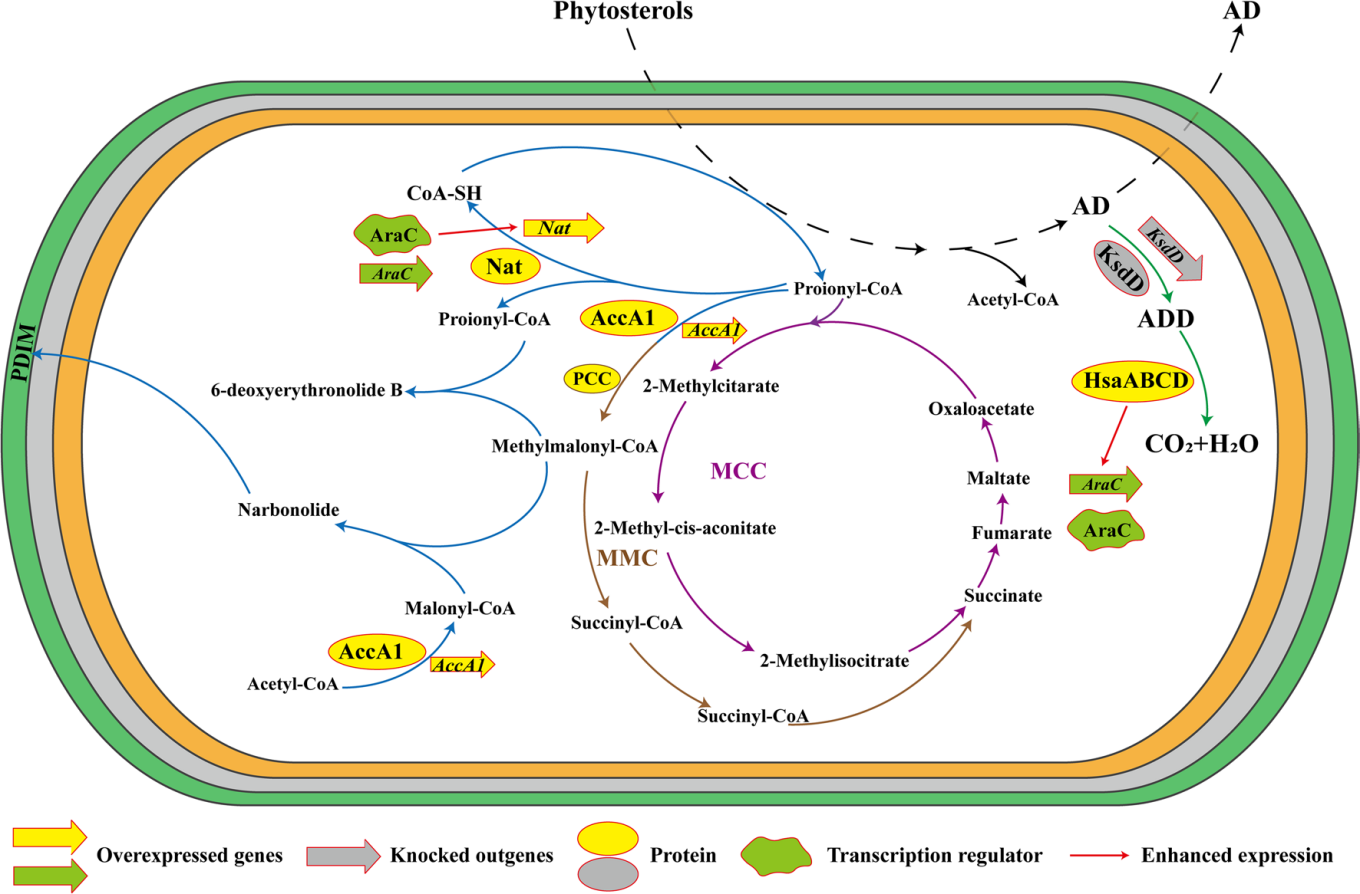
Pathways are used in combination to attenuate propionyl-CoA toxicity in MNR

Based on these mechanisms, this study shows for the first time that enhancement of the cytosolic methyl-branched lipid synthesis pathway with the *nat* operon can improve AD production in *Mycolicibacterium* (Fig. 1). The changes in cell growth and AD production by *AccA1* and *AccD1*, key genes of the cytosolic methyl-branched lipid synthesis pathway, and *nat*, the key gene of the *nat* operon, as well as the transcription factor AraC, were investigated. Meanwhile, to further improve AD production, the two pathways were jointly enhanced to construct a tandem expression strain, and the expression of the key gene *AccA1* was regulated by modifying the existing plasmid using the tetracycline operator according to the growth status of the bacteria. The resulting optimal strain achieved a 96.88% AD transformation rate. The multi-pathway combination addressed the toxic effects of propionyl-CoA for efficient AD production.

## Results and discussion

### Effect of enhanced cytosolic methyl-branched lipid synthesis pathway on biotransformation and strain physiology performances

#### Construction of cytosolic methyl-branched lipid synthesis pathway enhancing recombinant strains

Previous studies have shown the presence of AccA1 and AccD1 in MTB, and they are key enzymes in the cytosolic methyl-branched lipid synthesis pathway of the MTB [12]. The *AccA1* D174_19730 and *AccD1* D174_19735 were localized on the MNR VKM Ac-1815D (TAX:700508) genome by bioinformatics methods. Based on the functions of AccA1 and AccD1, their expression in MNR was aimed at enhancing the cytosolic methyl-branched lipid synthesis pathway and reducing the intracellular propionyl-CoA content. The recombinant strains QC M3-A and QC M3-D were successfully constructed according to the method described in 4.2, containing AccA1 and AccD1, respectively.

#### Influence of *AccA1* and *AccD1* enhancement on cell growth indicators and conversion indicators

**Fig. 2:**
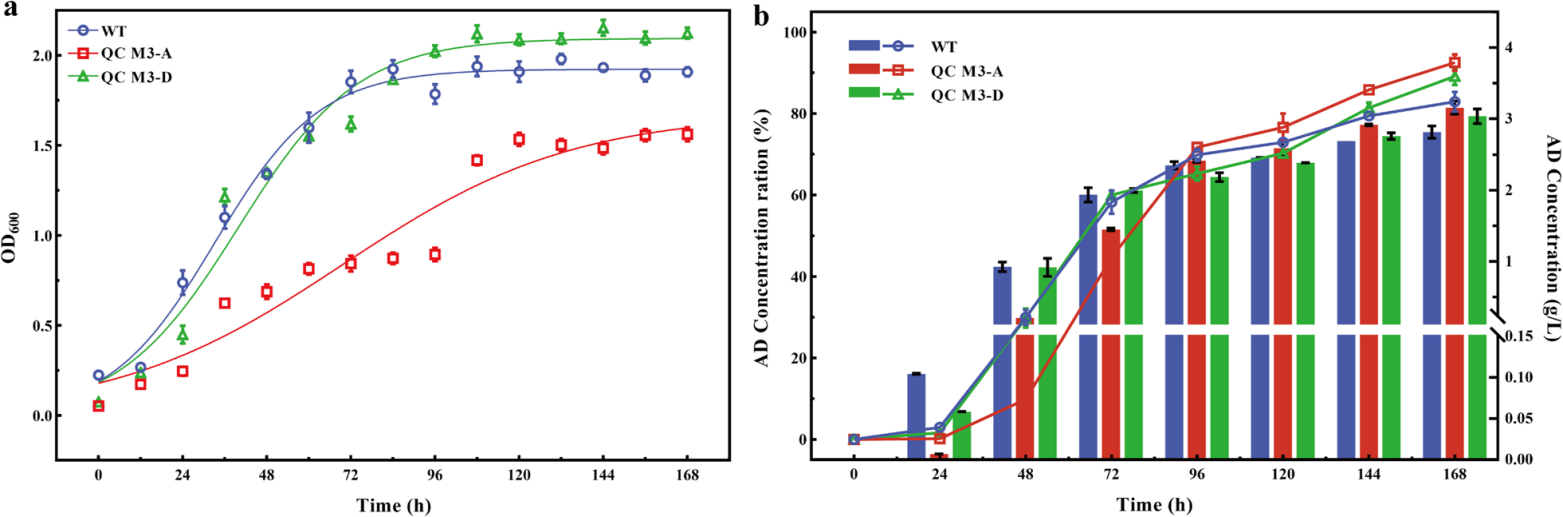
Recombinant growth status and AD conversion ratio of QC M3-A, QC M3-D. **a** Growth curves of WT, QC M3-A, and QC M3-D. **b** Time courses of phytosterol conversion ratio to AD by WT, QC M3-A, and QC M3-D. The folded line corresponds to AD concentration ration (left y-axis) and the bar corresponds to AD concentration (right y-axis). Error bars indicate the standard deviation from three independent experiments

The effects of *AccA1* and *AccD1* on cell growth were investigated and compared with the parental strains. All strains had typical growth curves, but the recombinant strain QC M3-A was severely affected and its biomass was lower than WT, with QC M3-A being the most severely affected (Fig. 2a). This is due to the over-expression of AccA1 leading to accelerated synthesis of cell membrane methyl branched lipids in the pre-growth phase of the strain, which generates growth stress. Previously reported results may also support this phenomenon in the opposite direction [23, 24]. Although the above recombinant strains showed a decrease in biomass, the highest molar transformation rates of recombinant strains QC M3-A and QC M3-D were increased by 9.7% and 6.3%. Compared to the starting strain, AD accumulation was 3.19 ± 0.09 g/L and 3.08 ± 0.1 g/L under 5 g/L sterol conditions, respectively (Fig. 2b).

#### Influence of *AccA1* and *AccD1* enhancement on substrate tolerance of recombinant strains

**Fig. 3:**
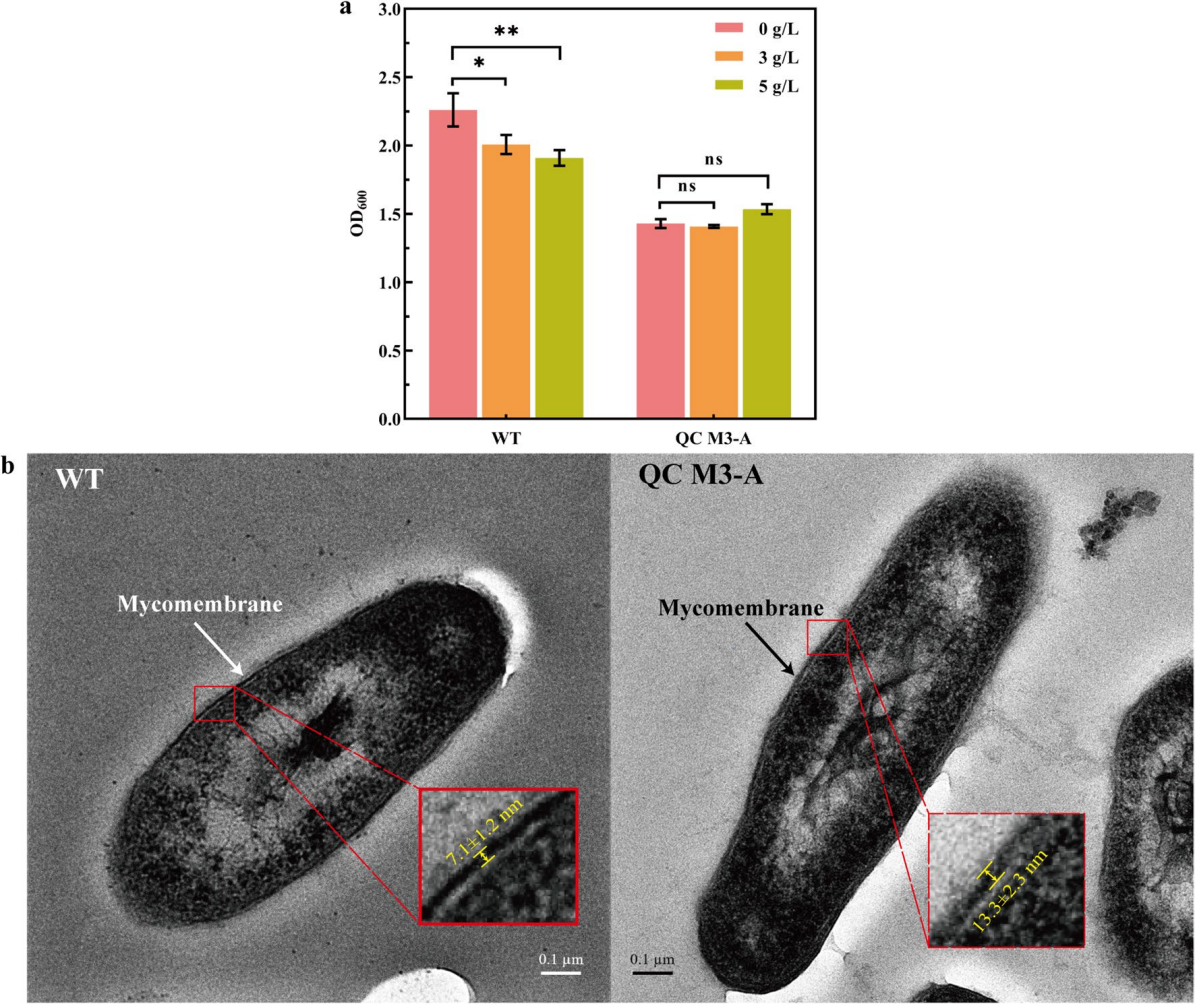
Growth status and cell structure. **a** Biomass (based on OD) of QC M3-A and WT at 120 h for different AD concentrations. **b** TEM image of QC M3-A and WT. Error bars indicate the standard deviation from three independent experiments and the levels of statistical significance are indicated as follows: *p < 0.05; **p < 0.01; ***p < 0.001; ns, indicates no significant difference (p > 0.05)

In the process of AD production by MNR, AD inhibits the growth and respiratory metabolic activities of the bacterium, and the gradually increasing AD concentration will produce more and more severe stress on the bacterium, which will severely inhibit its ability to convert sterols. To address this phenomenon, it was found that the intensity of AD production could be effectively improved by increasing the tolerance of the strain to AD [25, 26]. The growth of the strain was measured at different AD concentrations (Fig. 3a), and it was found that although the biomass of QC M3-A was lower compared to WT, it was less affected by AD concentration and showed better substrate tolerance compared to WT, which may be due to the overexpression of AccA1, which thickens the cell wall of the recombinant strain (Fig. 3b). The changes in these indicators are one of the reasons for the enhanced biotransformation capacity of QC M3-A.

### Reconstitution of the nat operon to promote intracellular acyl coenzyme A cycling

#### Construction of a nat operon overexpressing recombinant strain

**Fig. 4:**
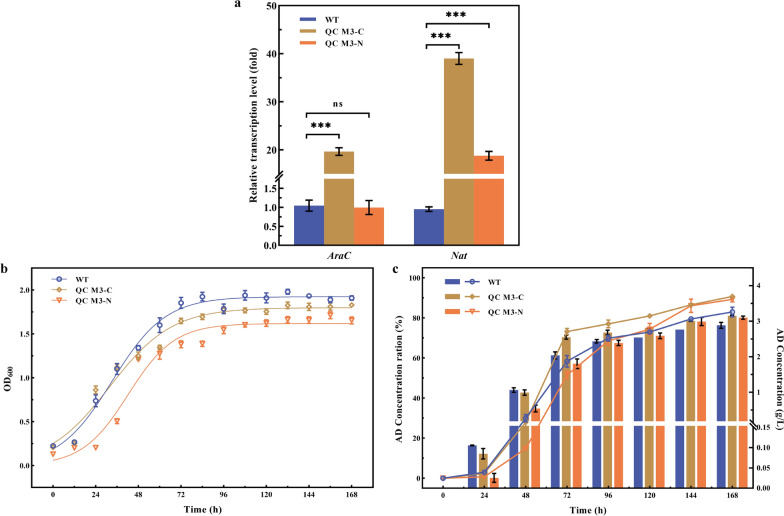
Recombinant strain qRT-PCR analysis, growth status, and AD conversion ratio of WT, QC M3-C and QC M3-N. **a** qRT-PCR analysis of the expression variations of *AraC* and *Nat*. **b** Growth curves of WT, QC M3-C and QC M3-N. **c** Time courses of biotransformation ratio of AD by WT, QC M3-C and QC M3-N. The folded line corresponds to AD concentration ration (left y-axis) and the bar corresponds to AD concentration (right y-axis). Error bars indicate the standard deviation from three independent experiments and the levels of statistical significance are indicated as follows: *p < 0.05; **p < 0.01; ***p < 0.001; ns, indicates no significant difference (p > 0.05)

In order to achieve a high yield of the target product AD, the AD downstream transforming *KsdD* in the sterol metabolic pathway was knocked out so that the strain would not produce androsta-1,4-diene-3,17-dione (ADD), so the subsequent sterol parent nucleus degradation reaction would stop. In the presence of Hsa family genes, *Mycobacterium* accomplishes the complete degradation of the sterol parent nucleus. At the same time, the expression of Hsa family genes enhances the expression of the transcriptional regulator AraC [20, 27]. To reconstruct this operon and to investigate the role of *AraC* and *Nat* in sterol conversion, recombinant strains QC M3-C and QC M3-N were constructed, respectively. As shown in Fig. 4a, the transcriptional level of *AraC* in QC M3-C reached 19.64-fold higher than that of WT, and *Nat* transcription level increased nearly 40-fold. However, *Nat* did not show an upregulatory effect on *AraC* in QC M3-N. The regulation of the acyl-coenzyme cycle within the strain by the *AraC* gene relies mainly on the regulation of the expression of various downstream acyltransferases. The results of comparing the regulatory effect of *AraC* on *Nat* in different strains confirmed this conclusion that the expression of *AraC* does regulate the transcription of *Nat*, but no negative feedback regulation of intracellular *Nat* content on *AraC* was found for the time being.

#### Growth indicators and conversion indicators

The effect of *nat* operon enhancement on cell growth and AD transformation was investigated. It was found that although all strains had the same growth trend, the biomass of the recombinant strains was reduced (Fig. 4b). As shown in Fig. 4c, the AD production capacity of the recombinant strains was higher than that of the starting strains. Both QC M3-C and QC M3-N bio-maximal molar conversion ratios were higher than that of the starting strains, and AD accumulation was up to 3.13 ± 0.02 g/L and 3.08 ± 0.05 g/L. The above results indicated that the overexpression of the *nat* operon increased the AD production capacity of MNR, with the transcription factor *AraC* overexpression having the optimal effect.

### Effect of different pathways on intracellular propionyl-CoA

**Fig. 5:**
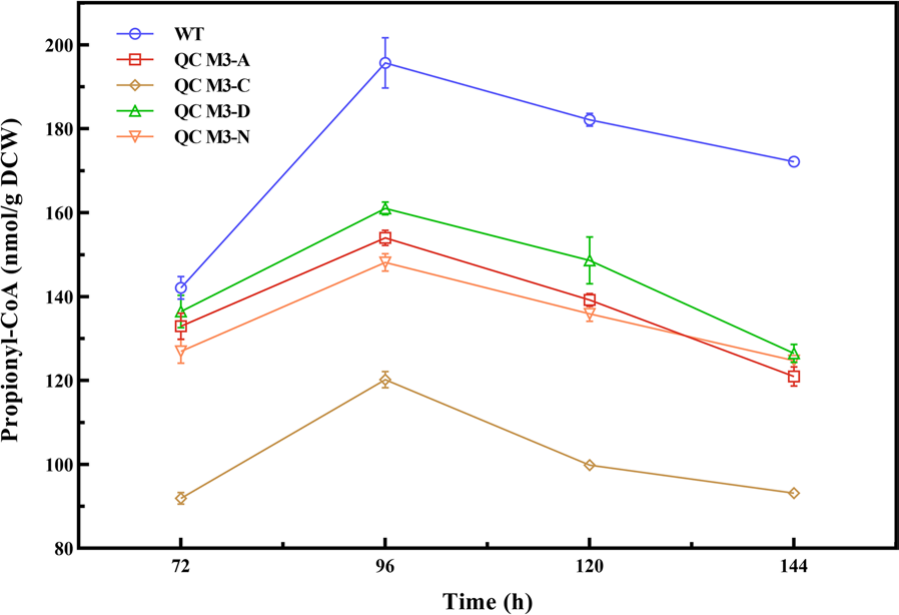
Intracellular propionyl-CoA levels in the biotransformation process of WT, QC M3-A, QC M3-C, QC M3-D and QC M3-N strains. Error bars indicate the standard deviation from three independent experiments

The intracellular propionyl-CoA levels of the recombinant strains in both pathways were examined (Fig. 5), and the results showed that the recombinant strains and the starting strains had similar trends of change. Meanwhile, the propionyl CoA content of all recombinant strains was lower than that of WT. Among them, QC M3-C had the lowest intracellular propionyl coenzyme A level, and its intracellular propionyl-CoA level decreased by 38.70% compared with the starting strain at 96 h of fermentation. At the same time, due to the overexpression of AraC, which accelerated the metabolism of intracellular propionyl-CoA and effectively promotes cellular coenzyme A circulation. This result could also well explain the better growth trend and AD productivity of QC M3-C relative to other recombinant strains. Combining the results of AD conversion rates of different strains in Figs. 2 and 4, we can find that all strains except recombinant strain QC M3-D had higher AD conversion rates than WT, although their biomass was lower than WT, which indicates that it is feasible to improve the conversion ability of strains by enhancing cytosolic methyl-branched lipid synthesis pathway and reconstitution of the nat operon.

### Combined enhancement of propionyl-CoA metabolism to improve transformation performance of strains

Three new recombinant strains were constructed to further enhance the metabolism of propionyl-CoA and to obtain a more productive and rapidly growing strain. Firstly, to address the problem of *AccA1* inhibition, the promoter element in the pMV261 plasmid was modified and its expression was regulated using the tetO operator to construct the overexpression strain QC M3-A_TetO_.

Based on the consideration of the slow growth of the strain, *AccA1* was expressed in tandem with *AraC* to construct the tandem expression strain QC M3-AC. *AraC* could significantly improve the viability of the strain, and in order to cope with the problem of inhibition of strain growth by *AccA1*, a strong RBS sequence (aaagaggtgaca) was added before the *AraC* in the construction of the tandem gene plasmid [28], hoping to use a high level of *AraC* expression to counteract the growth stress caused by *AccA1*. In addition, the two schemes were combined to construct a regulated tandem expression strain, so that on the one hand, *AraC* relies on the hsp60 promoter for constitutive expression, allowing the bacterium to maintain high viability throughout all stages of fermentation, and on the other hand, the *AccA1* is expressed in a regulated manner by the administration of an inducer at mid-fermentation, so that the two genes can work together at the highest intracellular propionyl-CoA level at mid-fermentation。

**Fig. 6:**
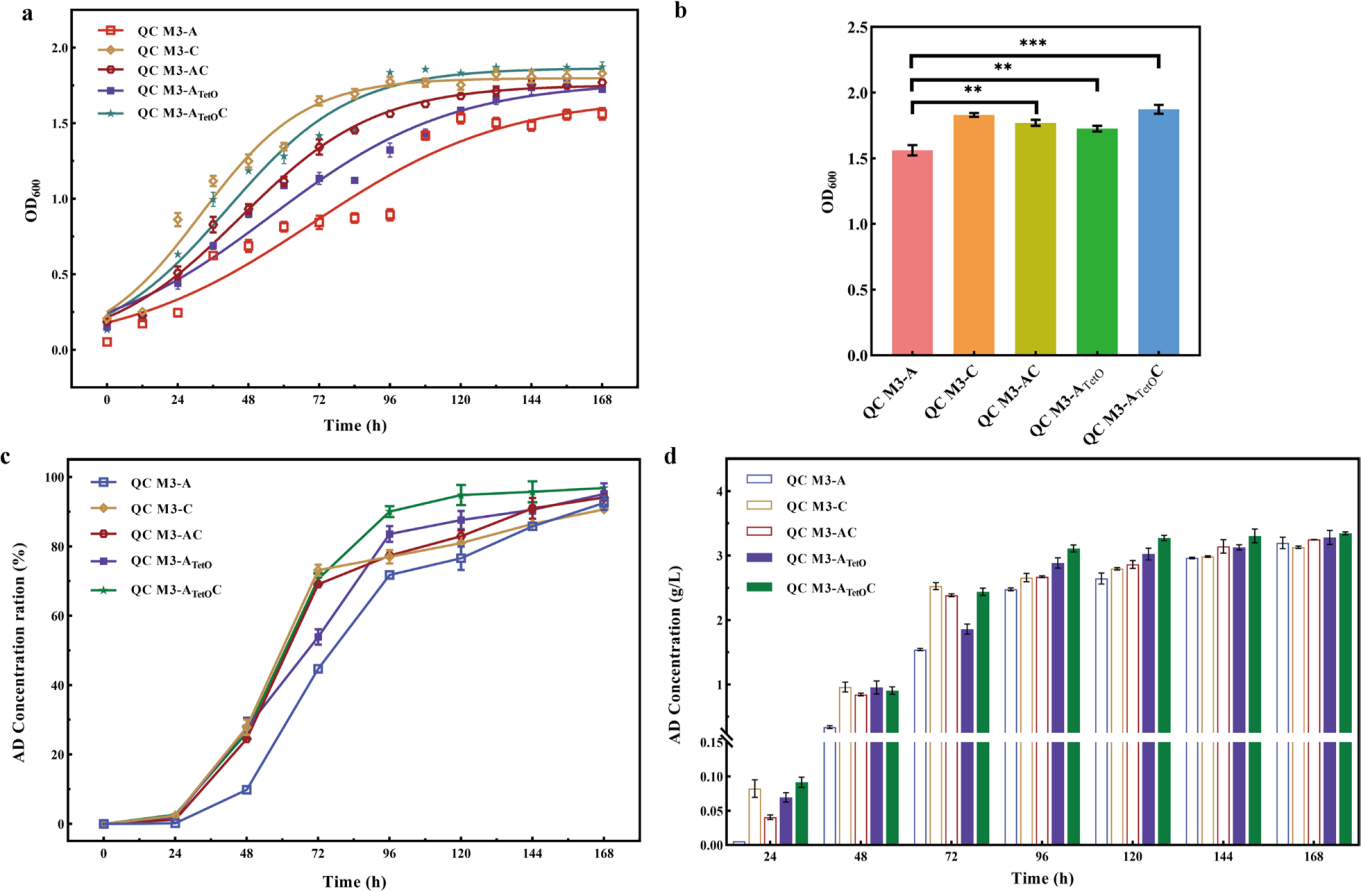
Recombinant growth status and AD conversion ratio of QC M3-AC, QC M3-A_teto_, QC M3-A_teto_C. **a** Growth curves of QC M3-A, QC M3-C, QC M3-AC, QC M3-A_teto_, QC M3-A_teto_C. **b** Biomass (based on OD) of QC M3-A, QC M3-C, QC M3-AC, QC M3-A_teto_, QC M3-A_teto_C at 120 h for different AD concentrations. **c** Time courses of phytosterol conversion ratio to AD by QC M3-A, QC M3-C, QC M3-AC, QC M3-A_teto_, QC M3-A_teto_C. **d** Time courses of AD concentration by QC M3-A, QC M3-C, QC M3-AC, QC M3-A_teto_, QC M3-A_teto_C. Error bars indicate the standard deviation from three independent experiments and the levels of statistical significance are indicated as follows: *p < 0.05; **p < 0.01; ***p < 0.001; ns, indicates no significant difference (p > 0.05)

As the data in Fig. 6a show, all three modes of tandem transformation strains, compared to QC M3-A strains can enter the logarithmic growth period quickly; in the late stage of fermentation, the biomass of the tandem strains were significantly increased, and remained basically the same as QC M3-C (Fig. 6b).

The tandem gene overexpression strains were constructed to improve the problem of the low AD conversion rate of *AccA1* overexpression strains in the pre-fermentation stage (Fig. 6a). As can be seen from Fig. 6c, all three tandem overexpression strains solved this problem well. Moreover, the conversion rate of the strains was improved with the addition of an inducer at 72 h. At 96 h, the AD conversion rate of QC M3-A_teto_C had reached 90.70%, which was close to the highest conversion rate of the single gene overexpression strain at 168 h and greatly shortened the conversion cycle. The bacterium showed excellent performance in AD conversion ability, and its highest AD conversion rate reached 96.88% at 168 h, which was 13.93% higher than that of the original bacterium WT, the final AD yield was 3.34 ± 0.02 g/L at a substrate concentration of 5 g/L. Meanwhile, the production rate was 1.3 times that of the original strain (Fig. 6c, Table 1). Compared to our previous work on propionyl-CoA regulation, we obtained similar or even higher production rate [6, 7]. The production efficiency of QC M3-A_TetO_C was lower than that of MNR-Fpcc-Fndh [6]. Although this phenomenon may be due to the different transformation systems, at the same time we speculate that this phenomenon may be due to the synergistic effect of simultaneous regulation of the two cofactors (NAD^+^/NADH and propionyl-CoA) in previous studies. Of course, in the subsequent work, we will also try to combine the regulation of multiple cofactors and explore the synergistic effect between cofactors to obtain higher conversion efficiency.

**Table 1:** Comparison of the impact of various regulation strategies on AD production

Strains	Regulation Strategies	Conversion yield (%)	Production rate (g/L/days)
QC M3-A	Overexpression *AccA1*	92.6	0.457
QC M3-D	Overexpression *AccD1*	89.2	0.44
QC M3-N	Overexpression *NAT*	89.3	0.441
QC M3-C	Overexpression *AraC*	90.7	0.447
QC M3-AC	Co-expression *AccA1* and *AraC*	94.1	0.464
QC M3-A_TetO_	Inducible regulation *AccA1*	95.1	0.469
QC M3-A_TetO_C	Inducible regulation *AccA1* and overexpression *AraC*	96.9	0.655
MNR-prpR [7]	MCC enhancement	90.6	0.628
MNR-prpDBC/ΔglnR [7]	*GlnR* knockout and MCC enhancement	94.3	0.654
MNR-Fpcc [6]	MMC enhancement	25.4	0.176
MNR-Fpcc-Fndh [6]	Overexpression *NDH-II* and MCC enhancement	96.4	0.669

## Conclusion

The aim of this study was to improve the AD production efficiency of the strain. A rational strategy was developed through genetic engineering and dynamic regulation methods. Overexpression of *AccA1, AccD1, AraC,* and *Nat* in MNR can reduce the intracellular propionyl-CoA content, while the combination of multiple pathways can better improve the transformation ability of the strain, which is an effective strategy to improve the catalytic performance of the microorganism. For the first time, the inducible regulation strategy by tet operator in MNR was used for biotransformation, which provided a new idea to further improve the AD production efficiency of the strain.

## Materials and methods

### Strains, plasmids, and culture conditions

All strains and plasmids used in this study are listed in Table 2, *E. coli* DH5α was used for plasmid construction. Strain MNR was stored in Tianjin University of Science and Technology Culture Collection Center (TCCC), Tianjin, China, and used for constructing engineering strains. *E.coli* was cultured in an LB medium. The culture method and medium of MNR were performed as previously described [29]. The medium of recombinant *Mycolicibacterium* strains was supplemented with kanamycin (50 μg/mL). The pMV261 plasmid was modified using primers to construct an expression regulatory plasmid with a double promoter and containing a tet operator [28, 30], named pMV262, and the expression of the target gene was regulated by the addition of tetracycline during fermentation.

**Table 2:** Strains, plasmids, and primers used in this study

Strains, plasmids, and primers	Significant properties	Source or purpose
Strains		
* Escherichia coli* DH5α	General cloning host	Transgen Biotech
M3	Wild type *Mycolicibacterium neoaurum* TCCC 11u978 (MNR)	Tianjin University of Science and Technology Culture Collection Center (TCCC)
QC M3	Deletion of *KsdD* in *Mycolicibacterium neoaurum* TCCC 11u978	Lab of Systematic Microbiology and Biomanufacturing Engineering, Tianjin University of Science and Technology
QC M3-N	QC M3 containing plasmid pMV261-*Nat*	This work
QC M3-C	QC M3 containing plasmid pMV261-*AraC*	This work
QC M3-A	QC M3 containing plasmid pMV261-*AccA1*	This work
QC M3-D	QC M3 containing plasmid pMV261-*AccD1*	This work
QC M3- AC	QC M3 containing plasmid pMV261-*AraC*-*AccA1*	This work
QC M3-A_TetO_	QC M3 containing plasmid pMV262-*AccA1*	This work
QC M3-A_TetO_C	QC M3 containing plasmid pMV262-*AccA1*-*AraC*	This work
Plasmids		
pMV261	Shuttle vector, *hsp60*, *Kan R*	Dr. W. R. Jacobs Jr
pMV262	Shuttle vector, *Tet O*, *Tet R*, *Kan R*	This work
pMV261-*Nat*	*Nat* was connected to pMV261	This work
pMV261-*AraC*	*AraC* was connected to pMV261	This work
pMV261-*AccA1*	*AccA1* was connected to pMV261	This work
pMV261-*AccD1*	*AccD1* was connected to pMV261	This work
pMV262-*AccA1*	*AccA1* was connected to pMV262	This work
pMV261-*AraC-AccA1*	*AraC* and *AccA1* were Series connected to pMV261	This work
pMV262-*AraC-AccA1*	Adding *tet* operon between *AraC* and *Acca1* at pMV262-AraC-AccA1	This work
Primers		
* AccA1*-f	gcggatccagctgcagaattcATGGTCAACGAACTCTTCCACAC	*AccA1* amplification
* AccA1*-r	tacgtcgacatcgataagcttTCATCGTTGGGACTCCTTGC	*AccA1* amplification
* AccD1*-f	gcggatccagctgcagaattcATGACGCATCGTGAAGCGC	*AccD1* amplification
* AccD1*-r	tacgtcgacatcgataagcttTCACATCCTGAAGACGCCGT	*AccD1* amplification
* Nat*-f	gcggatccagctgcagaattcATGACCGTCGATGTGGCCG	*Nat* amplification
* Nat*-r	tacgtcgacatcgataagcttTCAGTTCCCCAGCACGCG	*Nat* amplification
* AraC*-f	gcggatccagctgcagaattcGTGAATGCCCCTCGGCGT	*AraC* amplification
* AraC*-r	tacgtcgacatcgataagcttTCAGTTACCTCTCATCCATTCCAG	*AraC* amplification
* TetO*-f	ccgtggcgcggccgcggtaccGAATTCATAACTTCGTATAATGTATGCTATAC	*TetO* amplification
* TetO*-r	tcgaattctgcagctggatccCTGGATCCGCAATTGTCTTGG	*TetO* amplification
16S-RT-F	GTAGGGTCCGAGCGTTGTC	Quantitative RT-PCR
16S-RT-R	GCGTCAGTTACTGCCCAGAG	Quantitative RT-PCR
* AraC*-RT-F	TCCTCACCGGAAGTGTTCTC	Quantitative RT-PCR
* AraC*-RT-R	ATACGAGCGAACTCATCGGT	Quantitative RT-PCR
* Nat*-RT-F	GTCGCTACCTGTGTGACGTG	Quantitative RT-PCR
* Nat*-RT-R	CTGTACGTCTTCACCACCGA	Quantitative RT-PCR

### Construction of recombinant strains

The method of constructing recombinant strains has been reported in previous studies [7]. *AccA1*, *AccD1*, *AraC* and *Nat* were cloned from QC M3 genome and inserted into linearized pMV261 to construct recombinant plasmids named pMV261-AccA1, pMV261-AccD1, pMV261-AraC and pMV261-Nat, respectively, and the recombinant plasmids were dropped into the dedicated QC M3 to obtain recombinant strains, QC M3-N, QC M3-C, QC M3-A and QC M3-D. The tandem strain was constructed by inserting the fragment *AraC* into the linearized pMV261-AccA1 using the same method as in previous studies to construct the tandem recombinant plasmid pMV261-*AraC-AccA1*, and finally obtain the recombinant strain QC M3-AC. By using pMV262, *AccA1* was regulated to construct pMV262-*AccA1* and recombinant strain QC M3-*AccA1*_TetO_. Based on this, strong RBS was selected to construct pMV262-*AraC-AccA1* and recombinant strain QC M3-A_TetO_C (Additional file 1: Fig. S1) [28].

### Determination and analytical methods

The desired product was extracted from the fermentation broth according to the previously described method, and propionyl-CoA was extracted from the cells and detected by HPLC [7]. Measurements were performed using an HPLC system equipped with a UV detector. Chromatographic separation was performed on a reversed-phase C18 column (4.6 mm × 250 mm, 5 μm). AD detection was performed using 80% methanol aqueous solution as mobile phase and buffer A (acetonitrile) and buffer B (100 mM ammonium acetate, pH 5.8) as mobile phases for the detection of propionyl-CoA. The molar product yields of androstenedione were calculated using the following equation: androstenedione molar yield % = (C_AD_/M_AD_)/(C_PS_/M_PS_) × 100%, where C_AD_ and C_PS_ are the product concentrations (g/L) of AD and phytosterol, respectively; M_AD_ and M_PS_ are the molar masses of AD and phytosterol, respectively.

The intracellular propionyl CoA content was calculated using the external standard method with reference to the method of Zhang et al. [7]. Calibration curves were plotted with propionyl CoA standard working solution, and sample propionyl CoA concentrations were calculated from the standard curves using the interpolation method. Finally, the relative concentration of intracellular propionyl coenzyme A was calculated based on the cell dry weight of each sample.

### RNA extraction and Real-time quantitative PCR (qRT-PCR) analysis

For qRT-PCR analysis, cells were cultured for 48 h and collected by centrifugation at 8000×*g* for 10 min at 4 °C. RNA isolation was performed according to the method described in our previous description [7]. qRT-PCR analysis was performed according to the previously described method. Primers for qRT-PCR are listed in Table 2. 16S rRNA gene messenger RNA (mRNA) levels were used as housekeeping genes (internal controls) to normalize sampling errors. Relative gene expression levels were calculated by comparing the Ct method (2^−ΔΔ^Ct method) [31].

### Transmission electron microscope observation of cell structure

Fixation and observation of MNR using transmission electron microscopy (TEM) [32]. Ultrathin sections (90 nm) were obtained using a Leica EM U27 ultrathin sectioning machine and a Leica EN KMR3, and the sections were stained and observed on a Hitachi HT7700 transmission electron microscope. The cell wall thickness measurement using ImageJ [33].

### Statistical analysis

Data represent the mean and standard deviation (SD) of three independent experiments. Student’s t-test were used to determine significant differences between the data. Differences between two groups were measured by the Student’s t-test. A p value less than 0.05 was defined statistically significant difference.

## Supplementary Information

**Additional file 1. Plasmid profiles of recombinant strains**

## Data Availability

All data for this study are included in this published article and its additional file.

## Abbreviations

AD: Androstenedione
ADD: Androsta-1,4-diene-3,17-dione
MNR: *Mycolicibacterium neoaurum*
MCC: 2-Methylcitrate cycle pathway
MMC: Methylmalonyl cycle pathway
MTB: *Mycolicibacterium tuberculosis*
ACC: Propionyl-CoA /acetyl-CoA carboxylase
PikA1: Polyketide synthase
PDIM: Phthiocerol dimycocerates
NAT: N-acetyltransferase
DCW: Dry cell weight
LB: Luria–Bertani
CoA: Coenzyme A
qRT-PCR: Quantitative Reverse Transcription-PCR
mRNA: Messenger RNA
HPLC: High-performance liquid
TEM: Transmission electron microscopy
