# Supplementary material for: Combined enhancement of the propionyl-CoA metabolic pathway for efficient androstenedione production in Mycolicibacterium neoaurum
Source: Microb Cell Fact. 2022 Oct 20;21:218. doi: 10.1186/s12934-022-01942-x (PMC9585753; doi:10.1186/s12934-022-01942-x)
Supplement: Supplementary file 1 — Additional file 1. Plasmid profiles of recombinant strains [file 12934_2022_1942_MOESM1_ESM.docx]

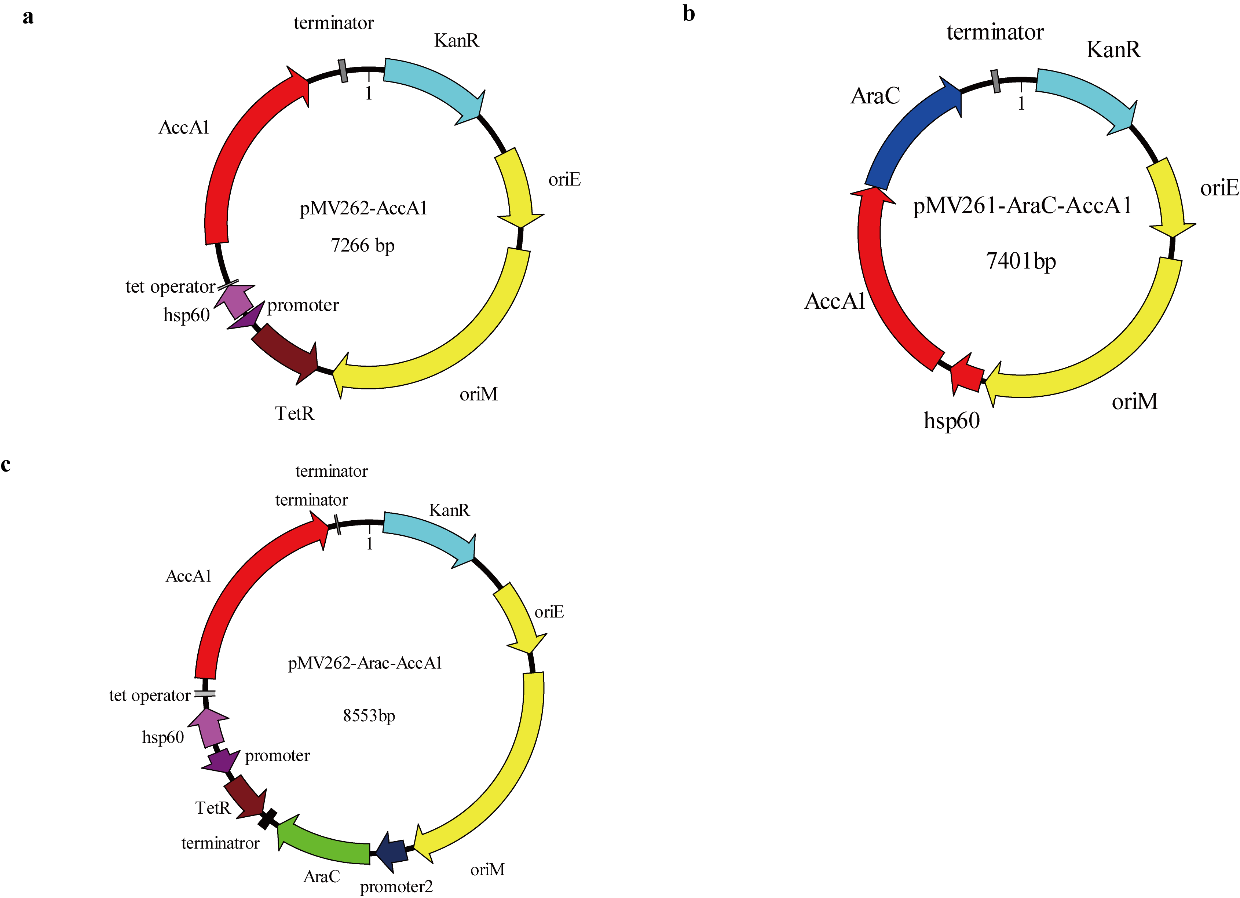


Supplementary Figure S1. Plasmid profiles of recombinant strains (a) pMV262-AccA1. (b) pMV261-AraC-AccA1. (c) pMV262-AraC-AccA1.
